# MDS-PB13 Score - Blood based detection of aberrancies by flow cytometry in patients with suspected and confirmed Myelodysplastic Neoplasms

**DOI:** 10.1038/s41375-024-02141-w

**Published:** 2024-01-16

**Authors:** Uta Oelschlaegel, Susann Winter, Katja Sockel, Katharina Epp, Jonas Schadt, Maximilian A. Röhnert, Thomas Krüger, Leo Ruhnke, Martin Bornhäuser, Uwe Platzbecker, Frank Kroschinsky, Malte von Bonin

**Affiliations:** 1 Department of Internal Medicine I, University Hospital Carl Gustav Carus, Faculty of Medicine Carl Gustav Carus, Technical University Dresden, Dresden, Germany; 2 National Center for Tumor Diseases (NCT), Dresden, Germany; 3 Clinic and Policlinic for Hematology, Cellular Therapy Hemostaseology and Infectiology, University Hospital Leipzig, Leipzig, Germany

**Keywords:** Myelodysplastic syndrome, Preclinical research

## To the Editor:

Flow cytometry (FCM) of bone marrow (BM) samples is part of the integrated diagnostic approach for myelodysplastic neoplasms (MDS) [1–3]. Several FCM-based scoring systems e.g. the Ogata-score and integrated FCM-score (iFS) have been implemented to explore dyspoiesis in maturing cells and myeloid progenitor cells (myPCs), the latter being important in FCM-diagnostics of MDS in BM [3–6].

To date, only a few studies with a limited number of patients and/or restricted antigen panels reported the use of blood to detect MDS-related aberrancies in patients with cytopenias and suspected MDS [7–10]. Aires et al. conducted a small pilot study in which granulocytes and monocytes were analyzed to develop a FCM-based scoring system [7]. Their approach examined FCM-aberrancies described partly by Cherian et al. and Rashidi et al., such as decreased side scatter and CD10 expression on granulocytes [8, 9]. Wagner–Ballon and the European LeukemiaNet International MDS Flow Working Group (ELN-iMDS-Flow-WG) validated the quantification of the classical monocyte fraction in blood as well as BM [10]. Meyerson and Alayed et al. introduced reduced expression of CD177 on granulopoiesis in blood and BM as diagnostic criterion in clonal myeloid disorders including MDS [11, 12]. Besides the above-mentioned detection of aberrancies, monitoring of the dysregulated immune response has been proposed as an alternative strategy [13].

The present study was designed to develop a comprehensive, easy-to-use diagnostic antibody panel to evaluate aberrant antigen expression in blood not only in granulocytes and monocytes but also in myPCs with enough power to schedule subsequent diagnostic steps.

Our study comprised newly diagnosed patients with cytopenias suspected of MDS (cohort 1, *n* = 115), treated MDS patients (cohort 2, *n* = 74), and healthy blood donors (HD; *n* = 26) from the University Hospital Dresden (2021-2023). Standard diagnostic work-up in patients with unexplained blood cytopenias included cytomorphology, histopathology, cytogenetics, molecular profiling, and FCM. Patients with MDS were categorized according to the WHO2022 classification [1]. In 76 of 115 (66%) patients of cohort 1, MDS including CMML was diagnosed. IPSS-M [14] was available for 59 of 65 patients with MDS. The 39 patients (34%) of cohort 1 who did not meet the diagnostic criteria for MDS or other neoplasia were termed nonMDS-cytopenias. In cohort 2, 47 of 74 (64%) treated MDS patients were not in complete remission (nonCR-MDS), all others were in hematological CR (CR-MDS) [15]. The vast majority of the CR-MDS were after allogeneic transplantation. Patient characteristics are detailed in Supplementary Table 1. Informed consent was obtained from all participants. The study received approval from the Institutional Review Board (EK289112008) and was conducted in accordance with the Declaration of Helsinki.

Paired samples of blood and BM of all 189 patients (cohort 1 and cohort 2) and blood samples of HD were analyzed by FCM. The FCM-panel for blood analysis (Supplementary Fig. 1A) was designed based on the preliminary study results mentioned above [7–12]. Given their diagnostic significance in BM [3–6], we additionally evaluated myPCs. Overall, 23 FCM-parameters were analyzed in this 2-tube-8-color-assay. BM analysis was performed in parallel according recent ELN recommendations [3] and diagnostic MDS-scores (Ogata-score, iFS) were applied [4, 5]. Samples were acquired on a FACS-Canto2 cytometer (BD Biosciences, San Jose, CA). The staining procedure for blood and BM was identical (bulk lysis/stain/wash); instrument settings and quality controls were performed as described previously [6]. At least 250,000 events were acquired per tube. To conduct a valid data analysis at least 20 events per subpopulation were required. The gating strategy and corresponding plots are displayed exemplarily in Supplementary Fig. 1B, C. Descriptive statistics were done using GraphPad Prism 8.4.3. (GraphPad Software, San Diego, CA). R environment for statistical computing (R Foundation for Statistical Computing, Vienna, Austria) was applied fitting univariate logistic regression models. The quality measures (sensitivity and specificity) of each assay (single parameters as well as FCM-scores) were determined by comparison to the “gold standard”. For all FCM-measurements, this comparator was the diagnosis of MDS according to WHO2022 criteria. Sensitivity was true positivity (MDS by WHO2022 vs. MDS suggested by FCM-parameter/assay) and specificity was true negativity (nonMDS by WHO2022 vs. FCM-parameter/assay not suggestive of MDS). To compare the quality measures of each FCM-score the Akaike information criterion (AIC) was used. The AIC has no unit. Only identical data sets can be compared. The absolute value itself does not hold a specific interpretation. The most precise score has the lowest AIC value.

Reference values for each of the 23 FCM-parameters were determined based on blood samples from patients with nonMDS-cytopenias and HD (Table 1B). The focus was on a high specificity. The results for all 23 analyzed FCM-parameters are detailed in Table 1B. The most prominent aberrancies in samples of both patients’ cohorts were an increased percentage of myPCs and a decreased percentage of CD10-expressing granulocytes.

**Table 1:** (A) Sensitivity, specificity, accuracy (in %), and AIC of all evaluated MDS-scores (B) Investigated FCM-parameters, reference values, assignment to previously described FCM-scores.

(A)										
FCM-scores^1^	Threshold	Cohort 1				Cohort 2				HD
		(115 pts.: 76 newly diagnosed MDS; 39 nonMDS-cytopenias)				(74 pts.: 47 nonCR-MDS; 27 CR-MDS)				
		Sensitivity	Specificity	Accuracy	AIC	Sensitivity	Specificity	Accuracy	AIC	Specificity
Aires (original)	≥2	57	69	61	91	47	78	58	36	92
Aires (adapted)	≥3	38	92	57	85	30	96	54	33	100
Cherian	≥4	4	96	55	96	0	100	33	36	100
Meyerson	< 30%	52	76	61	91	44	100	56	37	91
Ogata	≥2	22	95	47	91	28	100	54	32	100
**MDS-PB13**	≥3	**61**	**79**	**67**	**81**	**70**	**81**	**74**	**32**	**100**
*iFS (in bone marrow)*	*C* = *MDS*	*88*	*79*	*85*	*49*	*79*	*67*	*74*	*32*	*-*

(B)												
FCM-parameters		Reference values^2^	FCM-scores^1^					Cohort 1		Cohort 2		HD
			Aires	Cherian	Meyerson	Ogata	iFS	Sensitivity	Specificity	Sensitivity	Specificity	Specificity
**Gran**	Gran (%)	36.0–77.0						41	56	50	78	91
	Lympho/Gran (ratio: %)	≤1.2					▐	28	74	21	81	96
	FSC (MFI)	≥150 000	▐					2	92	6	89	95
	SSC (MFI)	≥140 000	▐	▐				20	92	17	100	96
	**SSC (MFI-ratio: Gran/Lympho)**	**≥5.0**				▐	▐	**45**	**92**	**28**	**70**	**100**
	CD10 (MFI)	≥3 500	▐	▐				33	80	61	78	71
	CD11a (MFI)	≤10 000		▐				9	92	33	100	95
	**CD11b (MFI)**	**≥8 000**	▐				▐	**22**	**77**	**15**	**89**	**100**
	**CD13 (MFI)**	**≥15 000**	▐				▐	**22**	**95**	**15**	**89**	**100**
	**CD16 (MFI)**	**≥1 000**	▐				▐	**41**	**79**	**40**	**78**	**81**
	CD45 (MFI)	≥1 000	▐					0	100	0	100	100
	CD66 (MFI)	≤10 000		▐				28	76	11	100	100
	CD116 (MFI)	3000–9000		▐				13	100	6	100	95
	**CD10 (%)**	**≥90.0**						**49**	**77**	**62**	**78**	**96**
	**CD34 (%)**	**≤5.0**					▐	**3**	**87**	**2**	**100**	**100**
	**CD56 (%)**	**≤5.0**					▐	**8**	**97**	**4**	**96**	**100**
	CD177 (%)	≥30.0			▐			61	64	44	78	91
**Mono**	**classical/non-classical mono (%)**^2^	**≤94.0 /** ≥ **2.5**	▐					**39**	**87**	**38**	**89**	**88**
	**CD56 (%)**	**≤10.0**	▐				▐	**34**	**87**	**15**	**93**	**100**
**myPC**	**myPC (%)**	**≤0.10**				▐	▐	**51**	**88**	**59**	**89**	**100**
	**SSC (MFI-ratio: myPC/Lympho)**	**0.9–1.4**					▐	**10**	**94**	**10**	**93**	**96**
	**CD45 (MFI-ratio: Lympho/myPC)**	**4.0–7.2**				▐	▐	**27**	**88**	**32**	**89**	**88**
	**CD56 (%)**	**≤20.0**				▐	▐	**6**	**97**	**5**	**100**	**100**

The 13 FCM-parameters highlighted in bold in part B of this table are included in the final MDS-PB13 score and investigated in all samples, the other FCM-parameters were analyzed only in the first 98 samples (71 newly diagnosed patients with cytopenias, 27 MDS under treatment) and all healthy blood donors.

iFS-score, calculated after bone marrow investigation, was included in italics as a comparison to the above mentioned diagnostic scores investigating blood.

^1^For details of all mentioned FCM-scores see references [4, 5, 7–11]. Ogata-score and iFS are diagnostic scores for BM evaluation. Some parameters considering abnormalities of granulopoiesis, monopoiesis, and myPC are also included in this study for blood. As lymphatic progenitor cells are not present in blood, this parameter of the Ogata-score was not implemented in our MDS-PB analysis.

^2^Reference values are calculated as mean +/− 2 standard deviations and sporadicly adapted to obtain a maximum specificity. Wagner–Ballon et al. [10] developed and validated the increase of the percentage of classical monocytes and Aires et al. [7] included the decrease of non-classical monocytes in their score. For these two features original reference values are taken from the respective publications.

*FCM* flow cytometry, *pts*. patients, *CR* complete remission, *HD* healthy blood donors, *iFS* integrated flow score, *Gran* granulopoiesis, *Mono* monopoiesis, *myPC* myeloid progenitor cells, *Lympho* lymphocytes, *FSC* forward scatter, *SSC* sideward scatter, *MFI* mean fluorescence intensity, *BM* bone marrow, *AIC* Akaike Information Criterion.

Despite a low percentage of myPCs in the blood of HD (median: 0.03% of CD45+ leukocytes), we were able to analyze the myPC compartment in a remarkably large number of samples independent of disease status: in 92% of newly diagnosed MDS, 87% of nonCR-MDS, 87% of nonMDS-cytopenias, 82% of CR-MDS, and all HDs, respectively. Besides the percentage, the absolute count of myPCs (>10/µL) has been described as well to be of prognostic importance [16]. However, the sensitivity of the latter parameter was lower (43% and 49% in cohort 1 and 2, respectively), accompanied by only a slight increase in specificity (97% and 93%; data not shown). At least one aberrancy within the myPC compartment was detectable in 70% of newly diagnosed MDS and 68% of nonCR-MDS. The absence of any myPC aberrancy was associated with a very high specificity for the presence of nonMDS-cytopenias (85%) and HD (92%). Remarkably, a high concordance between blood and BM for the detection of aberrant myPCs could be shown (cohort 1: 76%, cohort 2: 80%; Supplementary Table 2). Collectively, these findings confirmed feasibility and importance of myPC analysis for MDS diagnostics in blood samples.

After an interim analysis that included the first 98 samples suspected for MDS and all blood samples from HD we decided to omit 10 of the initial 23 parameters due to low sensitivity (mean fluorescence intensity (MFI) of FSC, SSC, CD10, CD11a, CD45, CD66, CD116) or low specificity (percentage of granulocytes, lymphocytes-to-granulopoiesis-ratio, MFI CD177). The final panel of 13 FCM-parameters (Supplementary Fig. 1A) demonstrated the best balance between sensitivity and specificity. It was applied to all 215 patients and HD (Table 1B).

Next, we calculated previously reported FCM-based blood scores and determined their quality measures (Table 1A). The Aires-score, which was developed for patients suspected of MDS using a threshold of two points [7], yielded a specificity of 69% analyzing nonMDS-cytopenias of cohort 1. With an adapted threshold of three points we observed a superior specificity (92%), but the sensitivity analysing newly diagnosed MDS decreased to 38%. The scores described by Cherian, Meyerson, or Ogata et al. [4, 8, 11, 12] showed a low sensitivity (4–52%).

**Fig. 1:**
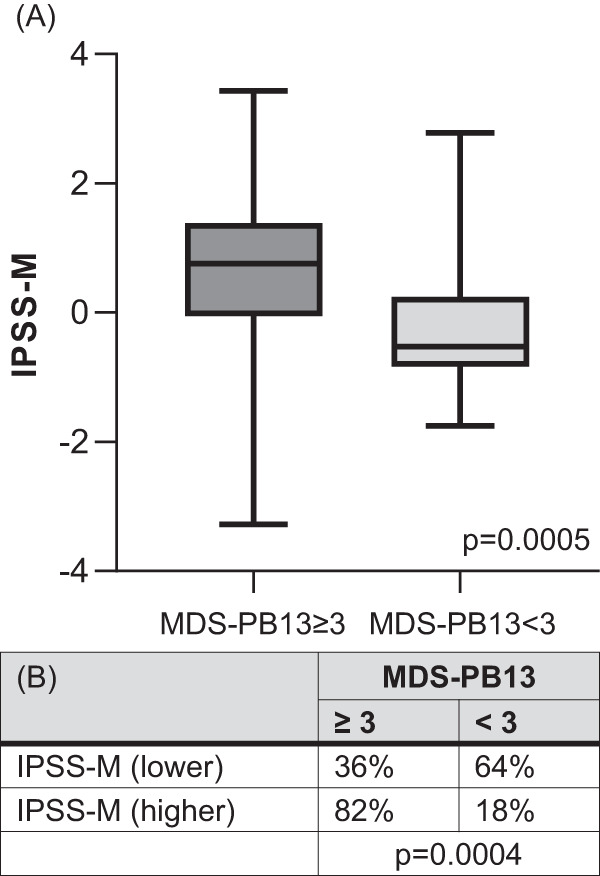
Association of MDS-PB13 score with IPSS-M in newly diagnosed MDS patients. IPSS-M was analyzed in accordance with Bernard et al. [14] and available for 59 treatment-naïve patients with a MDS diagnosis according to WHO2022 classification. **A** Box plot (median and IQR) of the IPSS-M risk score for patients with positive (≥3) and negative (<3) MDS-PB13 score (Mann–Whitney test). **B** Contingency table showing the frequency of patients with positive (≥3) and negative (<3) MDS-PB13 score in conjunction to lower (=very low / low / moderate low) and higher (=moderate high / high / very high) IPSS-M score (Fisher’s exact test).

The combination of the 13 FCM-parameters with the best diagnostic accuracy was termed MDS-PB13 score. For each aberrant FCM-parameter one point was added up. A sum-score ≥3 defined the threshold suggestive of MDS. Patients with newly diagnosed MDS or nonCR-MDS according to WHO2022 classification presented also with a MDS-PB13 score suggestive of MDS in blood in 61% and 70% of cases, respectively. In contrast, a sum-score ≥3 was rarely observed in samples from HD, nonMDS-cytopenias, and CR-MDS, resulting in a specificity of 100%, 79%, and 81%, respectively. The highest accuracy (67%) in combination with the lowest AIC (81) confirmed the new MDS-PB13 score to be the best-fitting model compared to the other FCM-scores. FCM-diagnostics performed in BM in parallel to blood revealed a better diagnostic power especially in cohort 1 patients. Detailed results of the various scores including the new MDS-PB13 score are depicted in Table 1A. These results suggest that the MDS-PB13 score might be a helpful, less-invasive tool in the diagnostic work-up in patients suspected for MDS. It remains to be shown whether the score can also be used for assessment of treatment response. In addition, the level of the MDS-PB13 score was correlated with the IPSS-M prognostic score. Significantly more patients with MDS-PB13 ≥ 3 presented with higher risk IPSS-M molecular abnormalities compared to patients without FCM-aberrancies. This was also reflected by significantly higher IPSS-M risk-level values in patients with MDS-PB13 ≥ 3 (Fig. 1A, B).

In 13 of 16 samples with poor quality BM aspirates (lack of BM crumps), MDS was still diagnosed by complementary methods (histopathology, cytogenetics, and mutational analysis). Importantly, FCM detected MDS-associated aberrancies in 12 of these BM samples and even in 9 of the blood samples applying the new MDS-PB13 score (Supplementary Table 3). Therefore, the detection of MDS-related aberrancies in blood is a valuable option also in these cases to point to MDS diagnosis.

In summary, we have developed a convenient FCM strategy, referred to as MDS-PB13 score, which combines the detection of FCM-aberrancies in different cell populations to suggest the presence of MDS in blood. Remarkably, the addition of myeloid progenitor cell analysis enhanced the diagnostic power. This score offers a preferable test reliability and as part of integrated diagnostics in blood it might represent a less-invasive tool to guide further diagnostic steps in patients suspected of having MDS. Thus, a MDS-PB13 score suggestive of MDS might speed up further BM diagnostics, whereas a BM aspiration might be less urgently required in patients with a low MDS-PB13 score. The MDS-PB13 score must be validated in a multicenter setting which is planned within the ELN-iMDS-Flow-WG. Furthermore, the potential use of the MDS-PB13 score for treatment monitoring and predicting patient outcomes, especially in the context of the genetic remission status, remains to be elucidated.

### Supplementary information

Supplementary Information to MDS-PB13 Score - Blood based detection of aberrancies by flow cytometry in patients with suspected and confirmed Myelodysplastic Neoplasms

## Data Availability

The datasets generated and/or analysed during the current study are available from the corresponding author upon reasonable request.
