# Supplementary material for: MDS-PB13 Score - Blood based detection of aberrancies by flow cytometry in patients with suspected and confirmed Myelodysplastic Neoplasms
Source: Leukemia. 2024 Jan 16;38(2):446–50. doi: 10.1038/s41375-024-02141-w (PMC10844065; doi:10.1038/s41375-024-02141-w)
Supplement: Supplementary file 1 — Supplementary Information to MDS-PB13 Score - Blood based detection of aberrancies by flow cytometry in patients with suspected and confirmed Myelodysplastic Neoplasms [file 41375_2024_2141_MOESM1_ESM.pptx]

## Slide 1
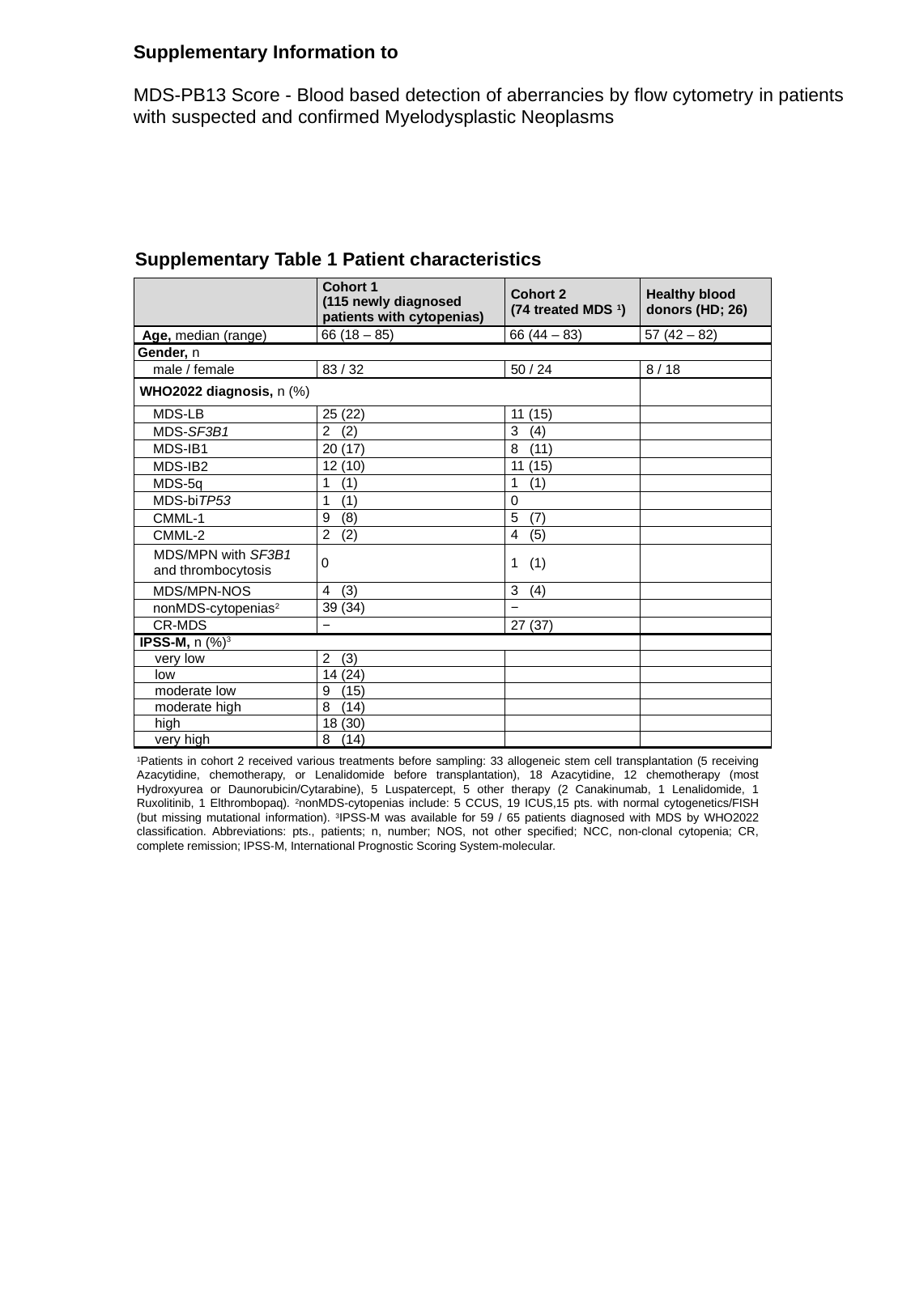

Supplementary Information to
MDS-PB13 Score - Blood based detection of aberrancies by flow cytometry in patients with suspected and confirmed Myelodysplastic Neoplasms
Supplementary Table 1 Patient characteristics
| | Cohort 1 (115 newly diagnosed patients with cytopenias) | Cohort 2 (74 treated MDS 1) | Healthy blood donors (HD; 26) |
| --- | --- | --- | --- |
| Age, median (range) | 66 (18 – 85) | 66 (44 – 83) | 57 (42 – 82) |
| Gender, n | | | |
| male / female | 83 / 32 | 50 / 24 | 8 / 18 |
| WHO2022 diagnosis, n (%) | | | |
| MDS-LB | 25 (22) | 11 (15) | |
| MDS-SF3B1 | 2 (2) | 3 (4) | |
| MDS-IB1 | 20 (17) | 8 (11) | |
| MDS-IB2 | 12 (10) | 11 (15) | |
| MDS-5q | 1 (1) | 1 (1) | |
| MDS-biTP53 | 1 (1) | 0 | |
| CMML-1 | 9 (8) | 5 (7) | |
| CMML-2 | 2 (2) | 4 (5) | |
| MDS/MPN with SF3B1 and thrombocytosis | 0 | 1 (1) | |
| MDS/MPN-NOS | 4 (3) | 3 (4) | |
| nonMDS-cytopenias2 | 39 (34) | − | |
| CR-MDS | − | ­­27 (37) | |
| IPSS-M, n (%)3 | | | |
| very low | 2 (3) | | |
| low | 14 (24) | | |
| moderate low | 9 (15) | | |
| moderate high | 8 (14) | | |
| high | 18 (30) | | |
| very high | 8 (14) | | |
1Patients in cohort 2 received various treatments before sampling: 33 allogeneic stem cell transplantation (5 receiving Azacytidine, chemotherapy, or Lenalidomide before transplantation), 18 Azacytidine, 12 chemotherapy (most Hydroxyurea or Daunorubicin/Cytarabine), 5 Luspatercept, 5 other therapy (2 Canakinumab, 1 Lenalidomide, 1 Ruxolitinib, 1 Elthrombopaq). 2nonMDS-cytopenias include: 5 CCUS, 19 ICUS,15 pts. with normal cytogenetics/FISH (but missing mutational information). 3IPSS-M was available for 59 / 65 patients diagnosed with MDS by WHO2022 classification. Abbreviations: pts., patients; n, number; NOS, not other specified; NCC, non-clonal cytopenia; CR, complete remission; IPSS-M, International Prognostic Scoring System-molecular.

## Slide 2
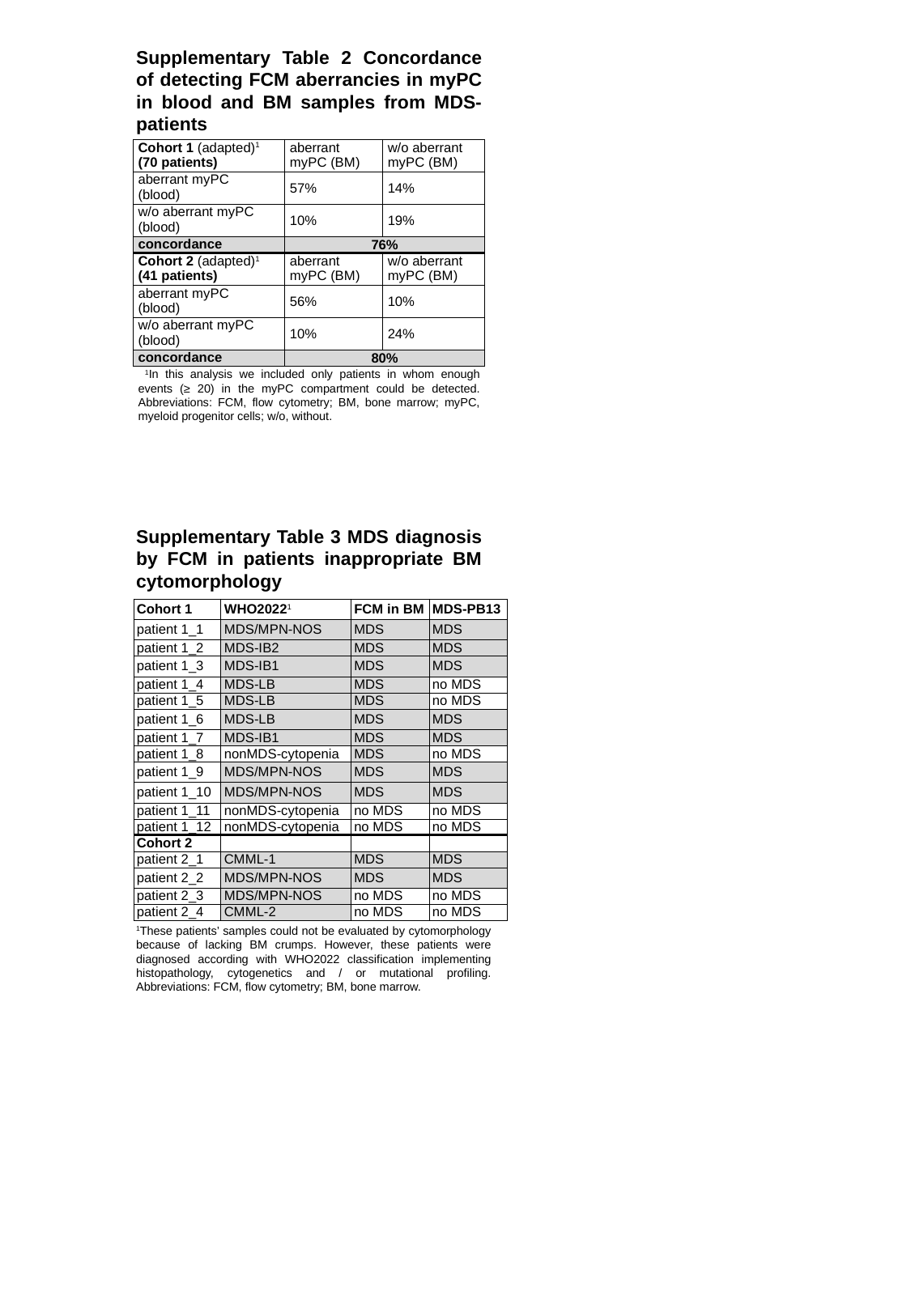

Supplementary Table 2 Concordance of detecting FCM aberrancies in myPC in blood and BM samples from MDS-patients
| Cohort 1 (adapted)1 (70 patients) | aberrant myPC (BM) | w/o aberrant myPC (BM) |
| --- | --- | --- |
| aberrant myPC (blood) | 57% | 14% |
| w/o aberrant myPC (blood) | 10% | 19% |
| concordance | 76% | |
| Cohort 2 (adapted)1 (41 patients) | aberrant myPC (BM) | w/o aberrant myPC (BM) |
| aberrant myPC (blood) | 56% | 10% |
| w/o aberrant myPC (blood) | 10% | 24% |
| concordance | 80% | |
 1In this analysis we included only patients in whom enough events (≥ 20) in the myPC compartment could be detected. Abbreviations: FCM, flow cytometry; BM, bone marrow; myPC, myeloid progenitor cells; w/o, without.
Supplementary Table 3 MDS diagnosis by FCM in patients inappropriate BM cytomorphology
| Cohort 1 | WHO20221 | FCM in BM | MDS-PB13 |
| --- | --- | --- | --- |
| patient 1\_1 | MDS/MPN-NOS | MDS | MDS |
| patient 1\_2 | MDS-IB2 | MDS | MDS |
| patient 1\_3 | MDS-IB1 | MDS | MDS |
| patient 1\_4 | MDS-LB | MDS | no MDS |
| patient 1\_5 | MDS-LB | MDS | no MDS |
| patient 1\_6 | MDS-LB | MDS | MDS |
| patient 1\_7 | MDS-IB1 | MDS | MDS |
| patient 1\_8 | nonMDS-cytopenia | MDS | no MDS |
| patient 1\_9 | MDS/MPN-NOS | MDS | MDS |
| patient 1\_10 | MDS/MPN-NOS | MDS | MDS |
| patient 1\_11 | nonMDS-cytopenia | no MDS | no MDS |
| patient 1\_12 | nonMDS-cytopenia | no MDS | no MDS |
| Cohort 2 | | | |
| patient 2\_1 | CMML-1 | MDS | MDS |
| patient 2\_2 | MDS/MPN-NOS | MDS | MDS |
| patient 2\_3 | MDS/MPN-NOS | no MDS | no MDS |
| patient 2\_4 | CMML-2 | no MDS | no MDS |
1These patients’ samples could not be evaluated by cytomorphology because of lacking BM crumps. However, these patients were diagnosed according with WHO2022 classification implementing histopathology, cytogenetics and / or mutational profiling. Abbreviations: FCM, flow cytometry; BM, bone marrow.

## Slide 3
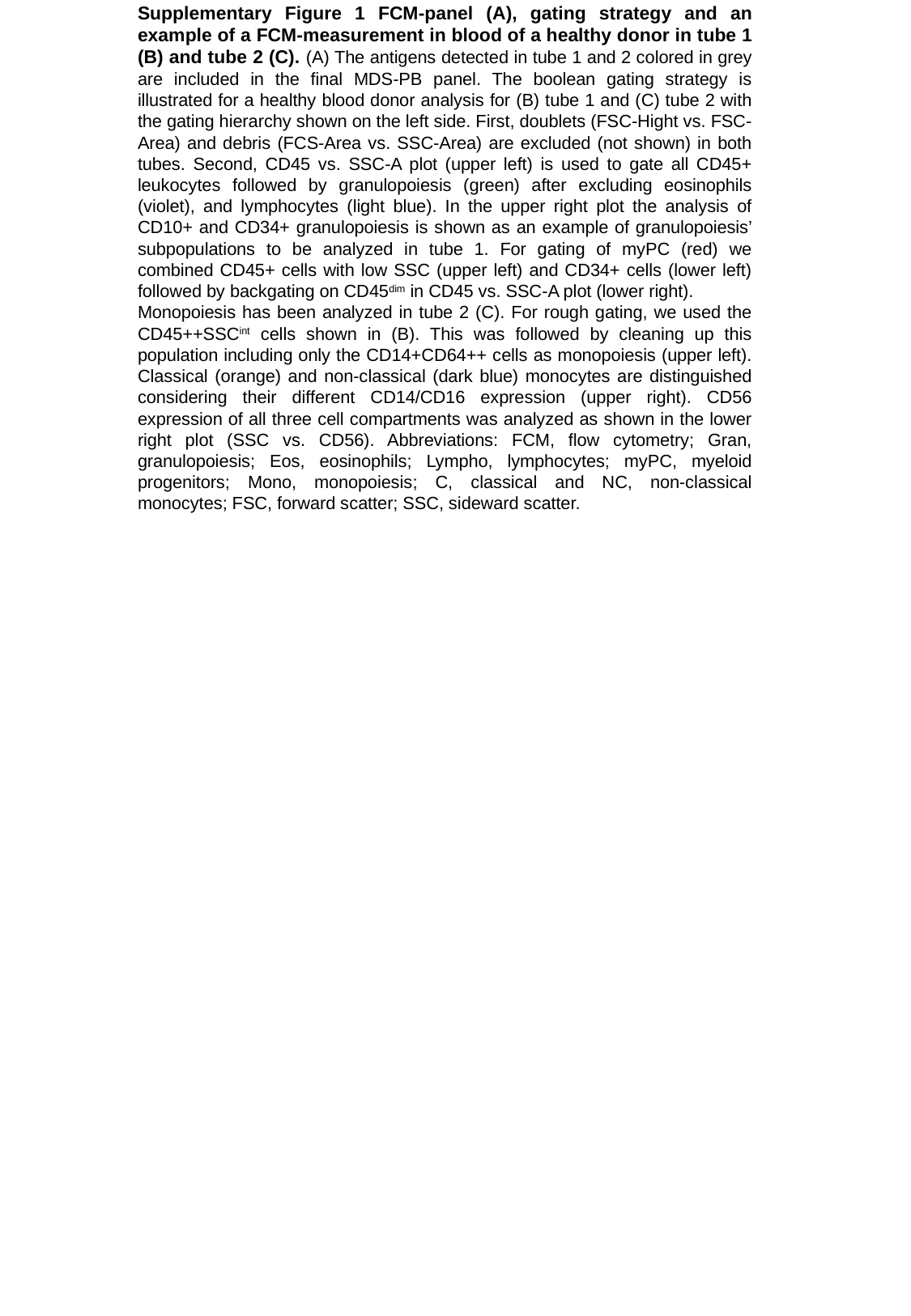

Supplementary Figure 1 FCM-panel (A), gating strategy and an example of a FCM-measurement in blood of a healthy donor in tube 1 (B) and tube 2 (C). (A) The antigens detected in tube 1 and 2 colored in grey are included in the final MDS-PB panel. The boolean gating strategy is illustrated for a healthy blood donor analysis for (B) tube 1 and (C) tube 2 with the gating hierarchy shown on the left side. First, doublets (FSC-Hight vs. FSC-Area) and debris (FCS-Area vs. SSC-Area) are excluded (not shown) in both tubes. Second, CD45 vs. SSC-A plot (upper left) is used to gate all CD45+ leukocytes followed by granulopoiesis (green) after excluding eosinophils (violet), and lymphocytes (light blue). In the upper right plot the analysis of CD10+ and CD34+ granulopoiesis is shown as an example of granulopoiesis’ subpopulations to be analyzed in tube 1. For gating of myPC (red) we combined CD45+ cells with low SSC (upper left) and CD34+ cells (lower left) followed by backgating on CD45dim in CD45 vs. SSC-A plot (lower right).
Monopoiesis has been analyzed in tube 2 (C). For rough gating, we used the CD45++SSCint cells shown in (B). This was followed by cleaning up this population including only the CD14+CD64++ cells as monopoiesis (upper left). Classical (orange) and non-classical (dark blue) monocytes are distinguished considering their different CD14/CD16 expression (upper right). CD56 expression of all three cell compartments was analyzed as shown in the lower right plot (SSC vs. CD56). Abbreviations: FCM, flow cytometry; Gran, granulopoiesis; Eos, eosinophils; Lympho, lymphocytes; myPC, myeloid progenitors; Mono, monopoiesis; C, classical and NC, non-classical monocytes; FSC, forward scatter; SSC, sideward scatter.

## Slide 4
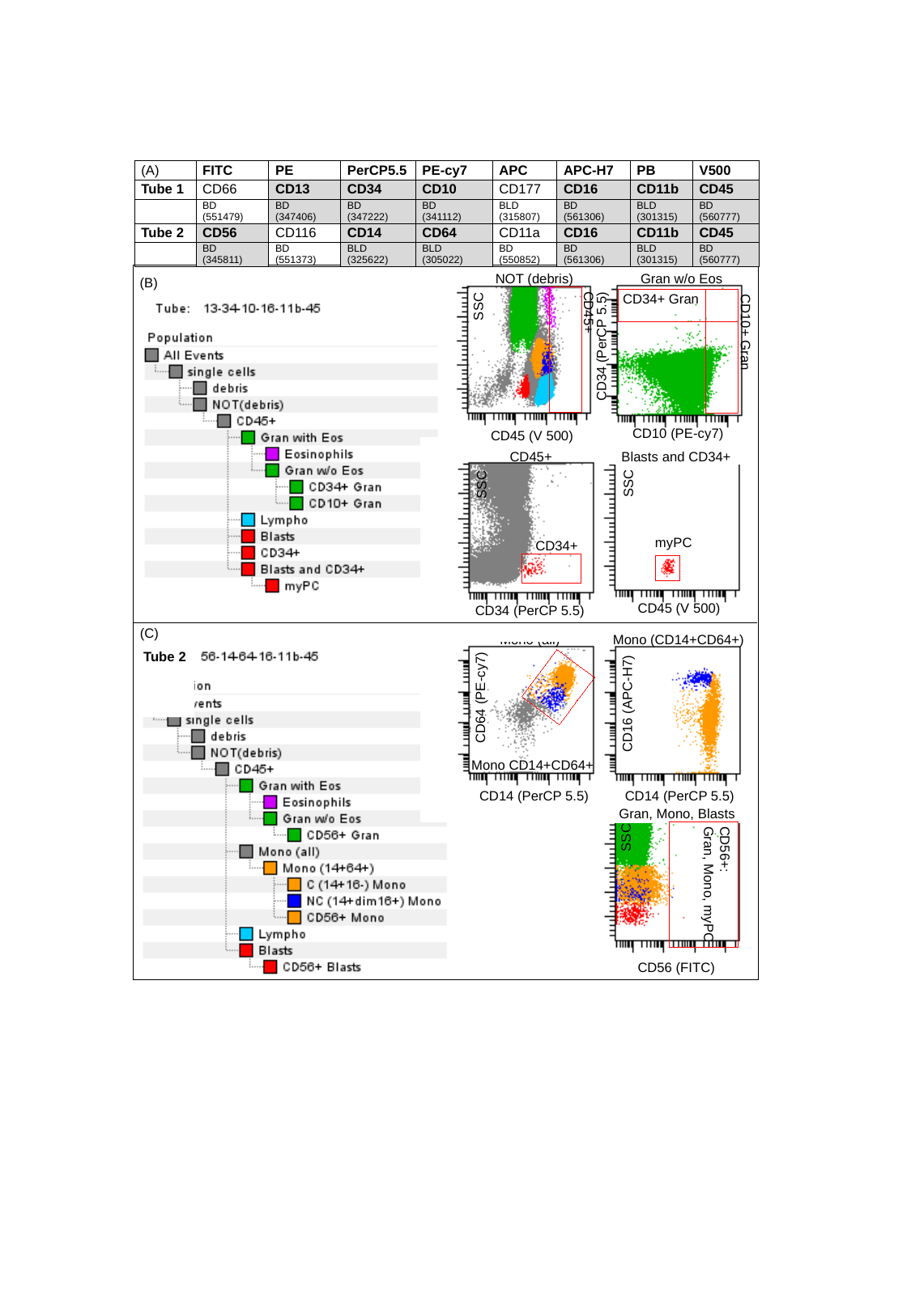

| (A) | FITC | PE | PerCP5.5 | PE-cy7 | APC | APC-H7 | PB | V500 |
| --- | --- | --- | --- | --- | --- | --- | --- | --- |
| Tube 1 | CD66 | CD13 | CD34 | CD10 | CD177 | CD16 | CD11b | CD45 |
| | BD (551479) | BD (347406) | BD (347222) | BD (341112) | BLD (315807) | BD (561306) | BLD (301315) | BD (560777) |
| Tube 2 | CD56 | CD116 | CD14 | CD64 | CD11a | CD16 | CD11b | CD45 |
| | BD (345811) | BD (551373) | BLD (325622) | BLD (305022) | BD (550852) | BD (561306) | BLD (301315) | BD (560777) |
NOT (debris)
Gran w/o Eos
(B)
CD34+ Gran
SSC
Tube 1
CD45+
CD10+ Gran
CD34 (PerCP 5.5)
CD10 (PE-cy7)
CD45 (V 500)
Blasts and CD34+
CD45+
SSC
SSC
CD34+
myPC
CD45 (V 500)
CD34 (PerCP 5.5)
(C)
Mono (CD14+CD64+)
Mono (all)
Tube 2
CD64 (PE-cy7)
CD16 (APC-H7)
Mono CD14+CD64+
CD14 (PerCP 5.5)
CD14 (PerCP 5.5)
Gran, Mono, Blasts
SSC
CD56+:
Gran, Mono, myPC
CD56 (FITC)
